# Immunological biomarkers of the vitreous responsible for proliferative alteration in the different forms of retinal detachment

**DOI:** 10.1186/s12886-020-01745-x

**Published:** 2020-12-28

**Authors:** Anikó Balogh, Tibor Milibák, Viktória Szabó, Zoltán Zsolt Nagy, Kai Kaarniranta, Miklós D. Resch

**Affiliations:** 1 Department of Ophthalmology, Semmelweis University, Mária u 39, Budapest, 1085 Hungary; 2 Department of Ophthalmology, Uzsoki Hospital Budapest, Uzsoki u. 29-41, Budapest, 1145 Hungary; 3 Department of Ophthalmology, University of Eastern Finland and Kuopio University Hospital, Kuopio, Finland

**Keywords:** Cytokine, Chemokine, Vitreous, Proliferative vitreoretinopathy, Retinal detachment, Proliferative diabetic retinopathy

## Abstract

**Background:**

The purpose of the study was to explore the immunological components that are responsible for the proliferative alterations in the different forms of retinal detachment (RD).

**Methods:**

Vitreous fluids were collected during 23G pars plana vitrectomy from 54 eyes of 54 patients with different RD types, such as rhegmatogenous RD (RRD) without proliferative vitreoretinopathy (PVR) (*n* = 30), PVR (*n* = 16) and proliferative diabetic retinopathy (PDR) with tractional RD (*n* = 8). Vitreous fluids were obtained from 19 eyes with epiretinal membrane (ERM), which were used as control samples. A multiplex chemiluminescent immunoassay was performed to evaluate the concentrations of 48 cytokines, chemokines and growth factors.

**Results:**

The expression levels of eotaxin, IFN-gamma, IL-6, IL-8, IL-16, MCP-1, MIF and MIP-1 beta were significantly higher in all RD groups than in the ERM group. The levels of CTACK, IP-10, SCGF-beta, and SDF-1 alpha were significantly higher in patients with diabetic tractional RD and PVR than in other patients. The upregulation of VEGF and IL-18 was detected in PDR.

**Conclusions:**

Our results indicate that complex and significant immunological mechanisms are associated with the pathogenesis of different forms of RD: selected cytokines, chemokines and growth factors are upregulated in the vitreous of eyes with RD. The detected proteins are present in different concentrations both in RRD and PVR. In the presence of PVR and PDR, the majority of cytokines are upregulated; thus, they may serve as biomarkers to estimate the progression or severity level of proliferation and later to develop personalized therapeutic strategies to slow down or prevent pathological changes.

## Background

Retinal detachment (RD) can cause vision loss if untreated, and even with proper surgical intervention, a potentially sight-threatening condition may develop in some cases. The most difficult challenges for vitreoretinal surgeons are proliferative diabetic retinopathy (PDR) complicated with tractional RD and proliferative vitreoretinopathy (PVR) developed from rhegmatogenous RD (RRD).

PVR is based on the development of fibrocellular membranes on the surface of and under the retina after RRD [1]. PDR is characterized by neovascularization on the retina and the formation of fibrovascular membranes at the vitreoretinal interface. The development of fibrovascular tissue often leads to hemorrhage and tractional RD [2].

According to our current knowledge, there is no cure or prophylaxis available for PVR yet, apart from the surgical approach [3]. In the treatment of PDR, pars plana vitrectomy plays the main role with various microsurgical techniques. Iyer et al. proposed a surgical algorithm for the management of PDR with tractional RD based on their compilation of relevant literature [4]. Because of these difficulties, the pathophysiology of PVR and PDR, including cytokines, chemokines and other inflammatory factors, is under investigation [5].

Many studies reported an immunological component responsible for PVR and the formation of tractional RD in PDR. In the first studies, only a few proteins could be assayed in one sample by enzyme-linked immunosorbent assay (ELISA) [6–8]. Caepaens et al. were among the first to evaluate three chemokines with ELISA in vitreous samples and found that the MCP-1 level was significantly higher in PVR and PDR samples than in controls [9].

Currently, a new technique, the multiplex bead-based immunoassay, provides an opportunity to perform a wide range of molecular analyses in one sample. This helps us understand the interaction among the components of the immunological processes responsible for pathological changes in PDR and PVR [10, 11]. Clinical evidence comparing intraocular cytokine, chemokine and growth factor levels in patients with PVR, PDR and RRD is scarce. Understanding the role of immunological factors in the pathophysiology of different RDs is important to be able to develop new therapeutic targets.

The purpose of this study was to explore the immunological components that are responsible for the proliferative alterations in PVR and PDR and to gain more detailed information and compare the differences in the levels of cytokines, chemokines and growth factors in the vitreous among the different forms of RD.

## Methods

The project was performed under the tenets of the Declaration of Helsinki after approval by the Hungarian Medical Research Council Committee of Science and Research Ethics (No. 15028–2/2017/EKU). All participants gave written informed consent to participate in the study.

Seventy-three eyes of 73 patients undergoing pars plana vitrectomy were included in the cross-sectional study. Patients were divided into four groups according to the indicated ocular pathology: 30 patients with RRD (without PVR), 16 patients with PVR, 8 patients with PDR and 19 control patients with idiopathic epiretinal membrane (ERM). The exclusion criteria were previous vitreoretinal surgery, penetrating injury, uveitis, aphakia, age-related macular degeneration, and uncontrolled glaucoma. Diabetes mellitus was excluded in the RRD, PVR and ERM groups. In the PDR group, only well-controlled diabetic patients (with glycated hemoglobin [HbA1c] under 8%) were included, and pure tractional RD was present (without vitreous hemorrhage or active proliferation). Previous panretinal photocoagulation was permitted, but intravitreal treatments (steroid or anti-VEGF) were excluded. A priori sample size calculation (power = 0.90; *p* = 0.05) was performed and provided the minimum number of eyes to be 65 eyes. The vitreous samples of patients with diabetic retinopathy, RD and ERM were collected for 2 years. As a limited number of samples can be analyzed on one kit of the multiplex chemiluminescent immunoassay, we had to define the maximum number of vitreous fluids. After we analyzed the patients’ data, we excluded the samples that were not matched the standing criteria. We had to exclude a lot of the diabetic samples because of previous intravitreal injections, active proliferation and high glycated hemoglobin.

Demographic and clinical data are summarized in Table 1.

**Table 1:** Demographic and clinical data of patients. Age, symptom duration and extent of RD are given in Mean ± SD. RRD - rhegmatogenous retinal detachment, PVR – proliferative vitreoretinopathy, PDR – proliferative diabetic retinopathy, ERM – Epiretinal membrane

		RRD	PVR	PDR	ERM
**N (male/female)**		30 (18/12)	16 (8/8)	8 (5/3)	19 (5/14)
**Age (years)**		61 (7,5)	58,4 (11,9)	55 (9,7)	70,7 (8,9)
**Symptom duration (days)**		7.0 ± 6.4	30.2 ± 28.3	43.4 ± 15.0	NA
**Macula on/off**		13/17	3/13	2/6	NA
**Extent of RD (quadrants)**		1.9 ± 0.7	2.9 ± 0.9	2.8 ± 0.8	NA
**Location of tears (%)**	**Superior**	50	31.2	NA	NA
	**Inferior**	6.6	56.3	NA	NA
	**Temporal**	36.7	0	NA	NA
	**Nasal**	6.6	12.5	NA	NA
**Endotamponade (%)**	**SF6 gas**	10	18.7	12.5	47.3
	**C3F8 gas**	73.3	50	50	52.7
	**Silicone oil**	16.7	31.3	37.5	0

### Sample collection

Pars plana vitrectomy (23G) was performed by two surgeons in two vitreoretinal centers. Before starting irrigation 0.5 ml undiluted vitreous samples were collected from the eyes. The samples were injected into sterile Eppendorf tubes, immediately cooled to − 20 °C then frozen at − 80 °C within 2 h and stored until the assay was performed.

### Measurement of cytokines, chemokines and growth factors in vitreous samples

The concentrations of cytokines, chemokines, and growth factors in vitreous samples were measured with a multiplex bead-based immunoassay: Bio-Plex system (Bio-Rad Laboratories, Hercules, CA, USA), molecules were detected with the Human Cytokine Screening Panel, 48-Plex (Bio-Rad Laboratories) after fourfold dilution. According to relevant previous works [12, 13]. The detailed specifications of the sample preparation were mentioned in our previous article [14]. Measurement of 84 molecules using 96-well assay plates and reagent kits included (see abbreviations): CTACK, eotaxin, basic FGF, G-CSF, GM-CSF, GRO-alpha, HGF, IFN-alpha2, IFN-gamma, IL-1 alpha, IL-1 beta, IL-1ra, IL-2, IL-2R alpha, IL-3, IL-4, IL-5, IL -6, IL-7, IL-8, IL-9, IL-10, IL-12/p40, IL-12/p70, IL-13, IL-15, IL-16, IL-17, IL-18, IP-10, LIF, MCP-1, MCP-3, M-CSF, MIF, MIG, MIP-1 alpha, MIP-1 beta, beta-NGF, PDGF-BB, RANTES, SCF, SDF-1 alpha, SCGF-beta, TNF-alpha, TNF-beta, TRAIL and VEGF were analyzed. If concentrations of the molecules were too low or too high, output was given as <OOR or > OOR meaning below or over the range (out of range).

### Statistical analysis

Cytokine concentrations were analyzed with, Kruskal–Wallis analysis of variance and Dunn’s multiple comparison test. *P*-values were calculated via dedicated statistical software (GraphPad Prism, La Jolla, CA). *P*-values < 0.05 were set to indicate statistical significance.

Exploratory factor analysis. Since not all correlations are well known among the examined cytokines, exploratory factor analysis was performed with SPSS 23.0 software (IBM, Chicago, IL, USA).

## Results

**Fig. 1:**
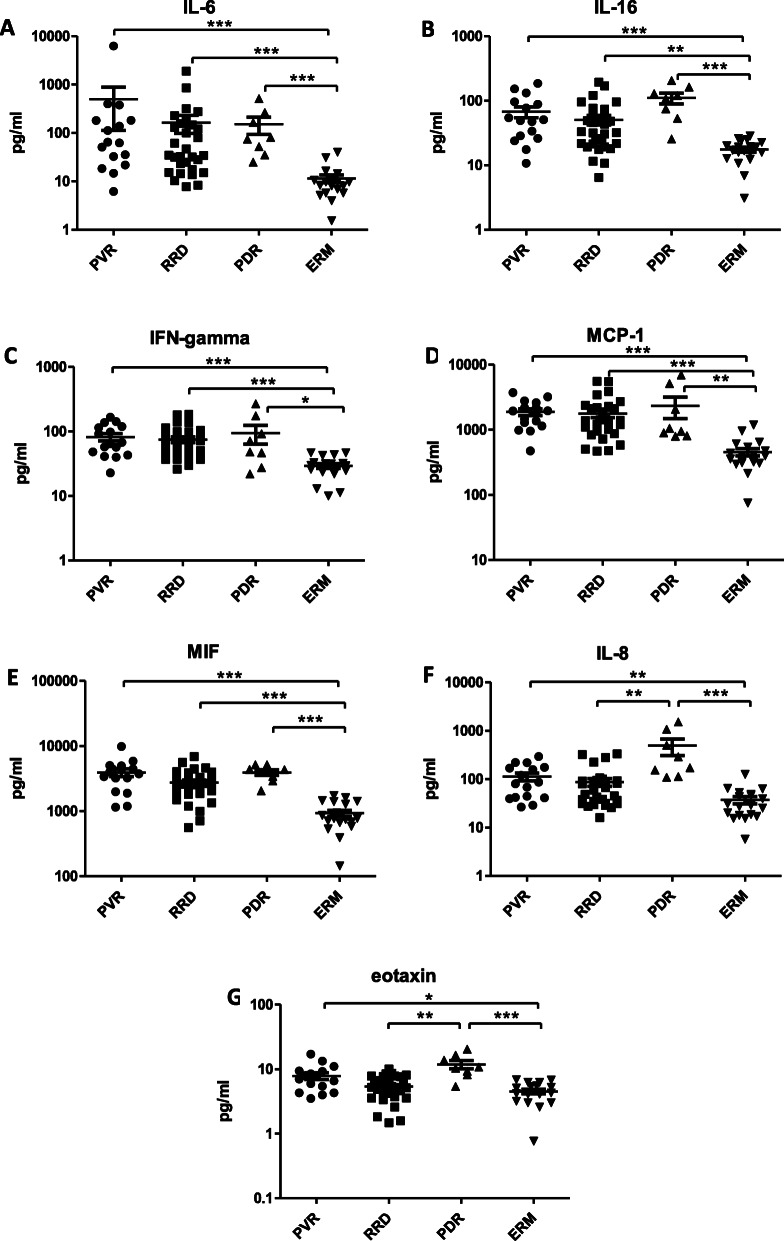
Upregulated molecules in PVR, RRD and PDR compared to ERM. Concentrations of IL-6, − 16, IFN-gamma, MCP-1, MIF, IL-8 and eotaxin in eyes with PVR, RRD, PDR and ERM. Statistically significant differences between the groups are marked by asterisks. * *p* < 0.05; ** *p* < 0.01; *** *p* < 0.001

**Fig. 2:**
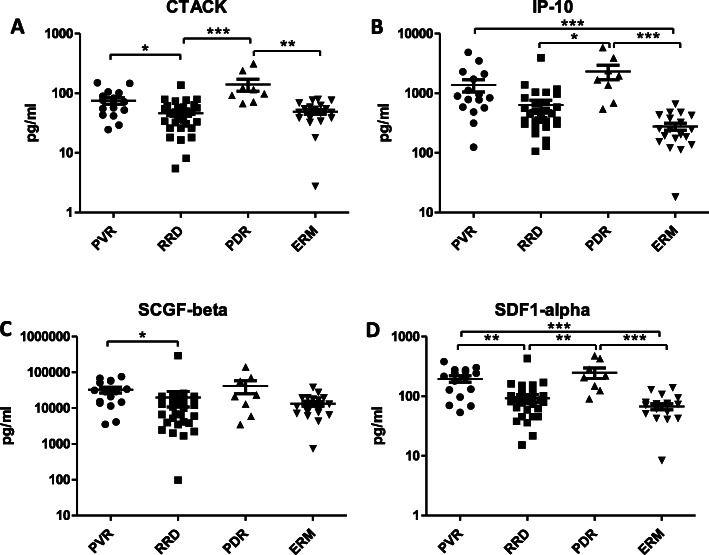
Upregulated molecules in PVR and PDR compared to RRD and ERM. Concentrations of CTACK, IP-10, SCGF-beta and SDF1-alpha in eyes with PVR, RRD, RPD and ERM. Statistically significant differences between the groups are marked by asterisks. * *p* < 0.05; ** *p* < 0.01; *** *p* < 0.001

**Fig. 3:**
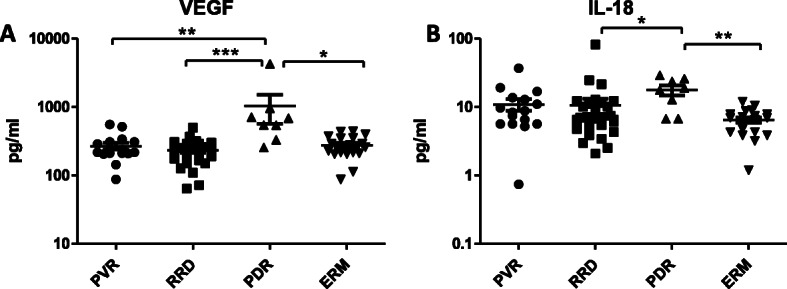
Upregulated molecules in PDR compared to PVR, RRD and ERM. Concentrations of VEGF and IL-18 in eyes with PVR, RRD, RPD and ERM. Statistically significant differences between the groups are marked by asterisks. * *p* < 0.05; ** *p* < 0.01; *** *p* < 0.001

**Fig. 4:**
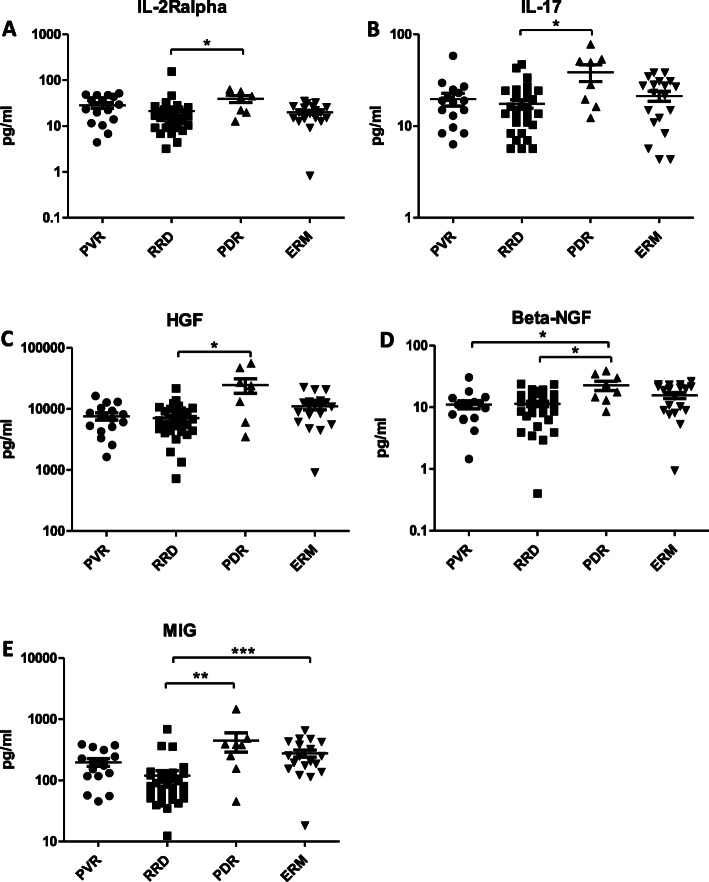
Upregulated molecules in PDR compared to RRD. Concentrations of IL-2Ralpha, IL-17, HGF, Beta-NGF, MIG in eyes with PVR, RRD, RPD and ERM. Statistically significant differences between the groups are marked by asterisks. * *p* < 0.05; ** *p* < 0.01; *** *p* < 0.001

An assay could be performed on all samples. Table 2 lists the median and SD of all individual cytokines in the four patient groups. The Kruskal-Wallis test selected 18 out of 48 cytokines that reached the level of significance in concentration (Table 3). The most important dependent variables are highlighted below in Figs. 1, 2, 3, 4.

**Table 2:** The concentration of cytokines in the groups in pg/ml (median; min.; max.)

	PVR			RRD			RPD			ERM		
	Median	Min	Max	Median	Min	Max	Median	Min	Max	Median	Min	Max
**CTACK**	69.32	24.56	149.7	44.84	5.47	137	106.9	67.46	313.3	47.89	2.76	79.3
**Eotaxin**	7.305	3.52	17.17	5.2	1.48	10.06	10.6	5.41	20.42	4.42	0.77	6.9
**Basic FGF**	426.8	202.1	1081	349.7	31.44	867.7	598.9	167.5	922.7	475.1	31.44	753.1
**G-CSF**	123.3	41.11	1103	100.1	37.46	263.4	126.6	48.31	1096	88.25	18.64	165.6
**GM-CSF**	3.62	0	13.74	4.64	0	13.88	5.87	2	15.29	5.79	0	11.87
**GRO-alpha**	163.7	0	308.9	168	124.7	434.8	152	0	387.7	152	0	244.7
**HGF**	7137	1635	16223	6208	720.8	21581	21941	3506	55875	10896	917.1	22566
**IFN-alpha2**	22.78	0.89	60.27	24.55	17.07	47.33	50.67	15	71.06	29.62	17.07	45.96
**IFN-gamma**	66.14	22.86	166.1	65.21	26.04	184.7	59.94	22.05	269.8	29.59	10.03	46.88
**IL-1alpha**	23.79	0.89	105.1	16.89	6.08	58.99	16.9	6.08	70.44	25.17	6.08	61.85
**IL-1beta**	3.5	1.42	11.39	3.64	1.42	9.32	3.78	2.33	12.03	4.34	1.73	8.4
**IL-1ra**	87.21	20.88	504.2	66.27	7.47	287.7	89.42	20.88	820.5	66.9	30.78	410
**IL-2**	9.735	3.12	27.86	6.745	1.42	26.15	9.945	5.68	29.99	10.59	3.98	18.47
**IL-2Ralpha**	26.41	4.4	51.97	15.71	3.21	154.1	43.65	12.73	62.66	19.87	9.16	35.33
**IL-3**	0.985	0.31	3.76	0.91	0.25	2.27	1.115	0.76	3.21	1.085	0.36	2.07
**IL-4**	1.6	0.41	4.04	1.6	0	3.2	2.06	0.83	4.86	1.97	1.03	2.5
**IL-5**	66.79	27.59	188.5	48.37	16.92	111	69.94	24.95	147.6	47.73	11.47	94.91
**IL-6**	63.49	6.2	6271	34.58	7.78	1899	78.36	25.09	514.9	9.77	1.54	40.17
**IL-7**	43.81	3.98	115.1	44.41	13.65	114.1	44.31	13.65	81.79	58.11	0	99.2
**IL-8**	83.66	26.62	295	54.07	15.98	336.2	232.2	109.6	1528	29.03	5.77	126.6
**IL-9**	17.37	5.73	36.96	13.99	5.22	30.68	17.63	7.79	47.47	14.25	5.73	28.58
**IL-10**	10.63	6.03	44.82	10.62	5.13	26.76	11.09	2.91	31.6	11.09	5.13	23.39
**IL-12(p70)**	17.66	5.61	79.77	16.17	5.61	54.04	17.29	4.06	68.36	21.36	6.38	46.84
**IL-12(p40)**	207.2	67.32	789.2	235.3	21.48	642.8	417.8	109.3	805.3	379.7	45.15	534.5
**IL-13**	2.28	1.07	5.13	1.93	0.76	3.57	3.165	1.36	9.08	1.93	0.44	3.83
**IL-15**	142.1	45.35	371.2	158.9	20.5	340.5	175	93.7	427.7	206	80.3	294.1
**IL-16**	50.1	10.74	185.5	32.52	6.49	193.3	114.4	25.68	206.2	17.13	3.1	28.67
**IL-17**	18.61	6.33	58.34	15.29	5.67	46.96	38.95	12.3	77.81	22.93	4.34	38.27
**IL-18**	8.555	0.74	36.88	6.65	2.08	81.89	18.34	6.76	29.11	6.99	1.19	11.92
**IP-10**	866.6	124.9	4869	433.4	106.1	3951	1827	548.8	5864	247.4	18.23	659
**LIF**	50.71	0	301.2	53.05	0	268	80.69	21.76	435.3	48.35	0.39	91.99
**MCP-1**	1865	475.1	3734	1361	472	5545	1005	794.9	6971	399.9	75.22	1192
**MCP-3**	4.16	0	12.2	3.53	0	8.44	3.88	0	46.28	4.6	0	7.99
**M-CSF**	27.35	8.69	54.58	18.99	6.15	108.3	24.21	10.37	55.82	22.54	4.45	46.69
**MIF**	3876	1150	9831	2550	560.7	6879	4156	2065	5183	780.3	144.6	1741
**MIG**	186.7	45.33	387.7	80.51	12.38	687.3	381.6	45.33	1467	247.4	18.23	659
**MIP-1alpha**	3.1	1	5.57	2.31	0.29	7.03	3.055	1	38.78	2.18	1	3.56
**MIP-1beta**	9.57	0	43.21	4.26	0	35.75	12.6	0	77.27	0	0	4.07
**beta-NGF**	11.01	1.45	30.51	10.79	0.4	23.79	20.48	8.54	38.77	18.88	0.94	26.32
**PDGF-BB**	75.19	52.71	180	76.22	52.71	124.3	138.7	78.27	181.7	98.09	61.51	140.5
**RANTES**	19.96	10.5	51.74	20.51	9.19	36.24	23.73	15.41	110.6	24.26	7.84	37.67
**SCF**	71.11	22.12	161.4	43.17	13.28	284	47.48	23.58	78.23	48.21	5.81	86.75
**SCGF-beta**	28963	3532	76005	10997	1684	295273	22418	3481	141845	11256	736.6	37990
**SDF-1alpha**	209.6	53.4	381.2	81.34	0	430.5	214.5	90.99	476.8	70.11	0	139
**TNF-alpha**	21.79	3.69	94.43	27.08	0	55.58	32.32	11.07	113.6	28.83	12.87	54.73
**TNF-beta**	6.73	0	45.18	5.52	0	30.32	9.7	0	38.86	7.93	0	27.07
**TRAIL**	13.45	3.44	29.53	13.19	6.78	25.51	18.38	5.68	112.4	10.01	0	21.96
**VEGF**	225	87.55	553.5	244.7	63.84	500.5	614.4	255.8	4289	272.1	87.55	443.4

**Table 3:** Cytokines with significant difference in case of RD. * *p* < 0.05; ** *p* < 0.01; *** *p* < 0.001

	RRD > ERM	PVR > ERM	PDR > ERM	PVR > RRD	PDR > RRD	PDR > PVR
**IL-6**	***	***	***			
**IL-16**	**	***	***			
**IFN-gamma**	***	***	*			
**MCP-1**	***	***	**			
**MIF**	***	***	***			
**IL-8**		**	***		**	
**eotaxin**		*	***		**	
**CTACK**			**	*	***	
**IP-10**		***	***		*	
**SCGF-beta**				*		
**SDF-1alpha**		***	***	**	**	
**VEGF**			*		***	**
**IL-18**			**		*	
**IL-2Ralpha**					*	
**IL-17**					*	
**HGF**					*	
**Beta-NGF**					*	*
**MIG**					**

Seven cytokines were upregulated in *all RD groups (RRD, PVR and PDR) compared to controls*: levels of IL-6 (*p* < 0.001, *p* < 0.001 and *p* < 0.001, respectively), IL-16 (*p* < 0.01, *p* < 0.001 and *p* < 0.001, respectively), IFN-gamma (*p* < 0.001, *p* < 0.001 and *p* < 0.05, respectively), MCP-1 (*p* < 0.001, *p* < 0.001 and *p* < 0.01, respectively), and MIF (*p* < 0.001, *p* < 0.001 and *p* < 0.001, respectively) were significantly higher in all RD groups than in the ERM group. The concentrations of IL-8 (*p* < 0.01, *p* < 0.001 and *p* < 0.01, respectively) and eotaxin (*p* < 0.05, *p* < 0.001 and *p* < 0.01, respectively) were significantly higher in PVR and PDR than in ERM and significantly lower in RRD than in PDR (Fig. 1). Further comparisons between groups are summarized in Table 3.

There were four upregulated cytokines in the *PDR and PVR groups compared to the RRD and ERM groups* (Fig. 2): CTACK was highly upregulated in patients with PVR (*p* < 0.05 PVR vs RRD) and PDR (*p* < 0.01 PDR vs ERM; *p* < 0.001 PDR vs RRD). Levels of IP-10 were upregulated in PDR and PVR vs ERM (*p* < 0.001 both) and upregulated in PDR vs RRD (*p* < 0.05); however, they were not different in PVR vs RRD. SCGF-beta exhibited the highest expression levels in PVR (*p* < 0.05 PVR vs RRD) but were not different in PDR vs ERM or RRD. SDF1-alpha was prominent in the PVR (*p* < 0.001 PVR vs ERM; *p* < 0.01 PVR vs RRD) and the PDR (*p* < 0.001 PDR vs ERM; *p* < 0.01 PDR vs RRD) groups.

The concentration values of VEGF in the vitreous fluid were significantly higher in the PDR group than in other groups (*p* < 0.05 PDR vs ERM; *p* < 0.001 PDR vs RRD and *p* < 0.01 PDR vs PVR). The vitreous level of IL-18 was found to be elevated in the PDR group compared to ERM (*p* < 0.01) and RRD (*p* < 0.05) (Fig. 3).

Levels of IL-2R alpha (*p* < 0.05), IL-17 (*p* < 0.05) and HGF (*p* < 0.05) were significantly higher in *PDR than in RRD*. The concentration of beta-NGF was significantly elevated in PDR compared to RRD (*p* < 0.05) and PVR (*p* < 0.05). The levels of MIG were significantly higher in PDR (*p* < 0.01) and ERM (*p* < 0.001) than in RRD (Fig. 4).

### Exploratory factor analysis

As the first step of exploratory factor analysis, variables (molecules) were filtered. Only 29 out of 48 molecules were included in the factor analysis, when valid concentration data were available for at least 70 out of 73 patients. Pearson correlation analysis revealed that a significant correlation was found among all 29 variables. The requirements of exploratory factor analysis were fulfilled: the communality value was higher than 0.3 among all variables, and the Kaiser-Meyer-Olkin (KMO) value was 0.833. The explanatory power of the 6 new variables was 82.36%; therefore, the dimension reductive method could be performed. The 29 variables could be grouped into 6 dimensions:

- Dimension 1: IL-15, IL-2, Basic FGF, IL-1 beta, IL-12 (p40), IL-7, IL-4, IL-17, RANTES, IL-5, IL-13 and GRO-alpha.
- Dimension 2: SCGF-beta, SCF, IL-2R alpha and M-CSF.
- Dimension 3: MIP-1 beta, MCP-3, IL-1ra, MIG and IL-8.
- Dimension 4: Eotaxin and CTACK
- Dimension 5: IL-18, MIF and IL-16
- Dimension 6: IFN-alpha2, PDGF-BB and TRAIL

Dimensions mean that the molecules within one dimension exhibit significant activity as individual biomarkers, and their changes are correlated.

**Fig. 5:**
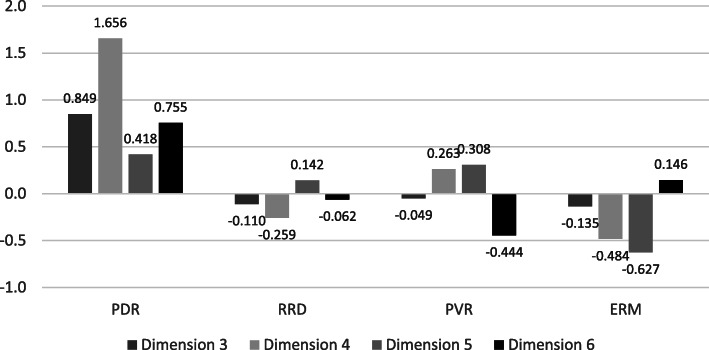
Exploratory factor analysis. Dimension 3: MIP-1beta, MCP-3, IL-1ra, MIG, IL-8; Dimension 4: eotaxin, CTACK; Dimension 5: IL-18, MIF, IL-16; Dimension 6: IFN-alpha2, PDGF-BB, TRAIL

Factor weights are summarized in Table 4, which shows that there is no difference among groups in dimensions 1 and 2, but significant differences were found in dimensions 3–6 (Fig. 5).

**Table 4:** Explorative factor analysis: Factor weights are summarized in the table, which shows that there is no difference among groups in dimensions 1 and 2, but significant differences were found in dimensions 3–6

		Component					
		1	2	3	4	5	6
Dimension 1	IL-15	0.915					
	IL-2	0.883					
	Basic FGF	0.881					
	IL-1beta	0.866					
	IL-12(p40)	0.840					
	IL-7	0.814					
	IL-4	0.765					
	IL-17	0.759					
	RANTES	0.730					
	IL-5	0.678					
	IL-13	0.626					
	GRO-alpha	0.494					
Dimension 2	SCGF-beta	-0.001	0.906				
	SCF	0.234	0.876				
	IL-2Ralpha	0.327	0.841				
	M-CSF	0.507	0.761				
Dimension 3	MIP-1beta	0.035	0.258	0.862			
	MCP-3	0.402	0.036	0.834			
	IL-1ra	0.252	0.053	0.783			
	MÍG	0.211	0.451	0.687			
	IL-8	0.052	-0.095	0.602			
Dimension 4	Eotaxin	0.233	0.130	0.014	0.812		
	CTACK	0.252	0.444	0.056	0.699		
Dimension 5	IL-18	0.132	0.209	0.098	0.033	0.824	
	MIF	-0.066	0.315	0.164	0.363	0.770	
	IL-16	-0.119	0.427	0.311	0.344	0.675	
Dimension 6	IFN-alpha2	0.466	0.043	0.148	0.244	0.057	0.727
	PDGF-BB	0.486	-0.037	0.228	-0.131	0.168	0.669
	TRAIL	0.249	0.092	-0.012	0.532	0.098	0.639

## Discussion

Our results show that common vitreous biomarkers involved in RDs are IL-6, IL-16, IFN-gamma, MCP-1 and MIF. This finding reveals the strong inflammatory component in the pathology of RD. However, different RD types show a phenotype-dependent profile in the expression of cytokines.

The interpretation of our data is challenging due to the complexity of the molecules and RD pathomechanism. Previous studies analyzed similar methods but different aspects of RD. Wladis et al. documented that IL-4, IL-5, IL-6, IL-15, G-CSF, GM-CSF, IP-10, MIP-1 alpha, SCF, and SCGF were significantly increased in PVR compared to primary RRD and ERM [15]. The upregulation of IP-10 and SCGF is in line with our observations. It seems that chemoattraction plays a central role in the pathogenesis of PVR when IL-8 and IP-10 are used as biomarkers. Upregulation of IL-6 and SCGF revealed that our PVR samples represent a late-state process with chronic inflammation and fixed retinal folds [16]. At present, there is no cure or effective prophylaxis for PVR. Autophagy regulators are effective in the prevention of PVR in animal models [17]. Pennock et al. suggested that ranibizumab might potentially prevent PVR formation since it reduces the bioactivity of the vitreous both in experimental animal models and humans. Ranibizumab prevented the growth of PVR membranes in rabbits as well [18]. In a mouse model, IL-6 receptor blockers have been shown to reduce retinal fibrosis [19]. Kawahara et al. proposed that statins might be potent inhibitors of cicatricial contraction in proliferative vitreoretinal diseases. In their paper they publsihed that in an experimental model the intravitreal injection of simvastatin dose-dependently prevented the progression of PVR [20]. Since acknowledgments of cellular signaling mechanisms have improved in recent years, we can develop specific targeted therapies to prevent PVR. For that purpose, patient material use in translational medicine research is crucial.

Abu El-Asrar et al. evaluated the levels of ten chemokines with ELISA in the vitreous from eyes with RRD, PVR, and PDR, and they came to the conclusion that MCP-1, IP-10, and SDF-1 might be factors in the pathogenesis of PVR and PDR. Our results are consistent with Abu El-Asrar’s, but we could analyze a wider range of molecules in each sample with the help of a multiplex bead-based immunoassay [8]. Wang et al. showed that levels of IL-6 and MCP-1 were significantly higher in the vitreous and aqueous humor in patients with PDR than in controls with macular hole (MH) [21]. Dai et al. documented that MCP-1, MIP-1 beta, IP-10, MIG and VEGF levels were increased in PDR compared to ERM and MH [22]. The same proteins were upregulated in the vitreous of our PDR samples. Moreover, CTACK and eotaxin levels were prominent in our vitreous samples. In PDR pathology, chemoattraction seems to be active, but as a disease characteristic sign, increased angiogenesis through VEGF can be observed, as previously shown. Note that elevated VEGF levels were not detected in PVR pathogenesis. The role of VEGF in diabetic macular edema and PDR is well known [23]. We observed that the levels of IL-18 and VEGF were significantly higher in PDR than in other conditions. Song et al. documented that the levels of intravitreal VEGF and IL-18 were significantly higher in active PDR than in ERM and MH [24]. Xu et al. found that the vitreous levels of CCL2, CXCL4, CXCL9, CXCL10, VEGF, sVEGFR-1, sVEGFR-2, IL-6, IL-8, IL-10, and IL-18 were elevated significantly in the PDR group compared to the nondiabetic group [25]. In our study, we found significant upregulation in the levels of IL-18 in PDR compared to the control group and RRD separately. Increased IL-18 expression levels in vitreous fluid reveal inflammasome activation [26]. Inflammasomes are large cytosolic protein complexes composed of Nod-like receptor sensor protein, adaptor protein ASC and caspase, mainly caspase-1, as an effector enzyme [27]. Inflammasome activation results in the release of the proinflammatory cytokines IL-1 beta and IL-18. Since inflammasomes seem to be activated during the late state of the DR process, they might be a good therapeutic target to prevent tractional RD once there is no response to current therapy drugs.

Takahashi et al. documented increased levels of IL-6, IL-8, IP-10 and MCP-1 in RRD compared to the control MH group [12]. These results are similar in our study when ERM cases were used as controls. They also found higher IL-6 and IL-8 levels, but not MCP-1 and IP-10, in RRD than in PDR. Our study showed higher IL-8 and MCP-1 levels in PDR than in RRD. This reveals a stronger chemoattraction in PDR with tractional detachment. Similar to our study, Pollreisz et al. found that IL-6, IL-8 and MCP-1 were elevated in the vitreous of RRD eyes compared to ERM. In contrast to our results, they observed an increase in IP-10 and MIP-1 alpha in RRD [28]. Rasier et al. found that the levels of IL-8 and VEGF were elevated in vitreous samples in eyes with RRD compared to samples from eyes with ERM and MH [29]. Ricker et al. reported that the concentrations of IL-1 alpha, IL-2, IL-3, IL-6, VEGF and ICAM are increased in the subretinal fluid of PVR but not in RRD [30]. We show here that the expression of VEGF was significantly higher only in the PDR group than in the RRD, PVR and ERM groups. Our results are consistent with previous reports [12, 15]. It seems that VEGF has the strongest biomarker role in PDR with and without tractional detachment. Interestingly, the levels of CTACK, IP-10 and SDF1-alpha were significantly higher in PVR and PDR than in RRD, while the stem cell factor SCGF had a stronger presence in PVR than in RRD. The concentration of these four chemokines was augmented only in the proliferative forms of RD. CTACK, IP-10 and SDF-1 play a role in a wide variety of processes, such as chemotaxis, immune response, cell-cell signaling, differentiation, activation of peripheral immune cells, and regulation of endothelial cell proliferation.

When we obtain the result of factor analysis, we focus on which factor and variable coheres with each other. At the same time, we can evaluate the factor weights of the variables to be able to hierarchize the variables of one factor. The bigger the factor weight the more important the variable of one factor, so we can analyze separately how the factor weight develops in a given factor. For example in the group of factor 3 MIP-1beta was the most important element, it had a bigger factor weight than IL-8 (Table 4).

To our knowledge, our report is the first to evaluate the concentrations of these 48 cytokines, chemokines, and growth factors in different forms of retinal detachment, including RRD, PVR and PDR with tractional RD. However, our study has some limitations, such as the complexity and high number of cytokines, that need further investigation to detect their relationships more precisely. RD and PDR present with variable clinical features, which might contribute to the multiplex variations in cytokines in fluids. In addition, it cannot be identified whether cytokines are upregulated in the vitreous due to RD (as a consequence) or are already present before detachment (as a causative agent). This limitation is hard to solve due to ethical reasons since the vitreous of healthy human eyes is not accessible in everyday routine clinical care. The exploratory factor analysis highlights some new possible connections and relations among cytokines, which can show some further directions of investigation.

## Conclusions

We conclude that our results indicate that complex and significant immunological mechanisms are associated with the pathogenesis of different forms of RD, such as RRD, PVR and PDR. Cytokines, chemokines and growth factors are upregulated in the vitreous of eyes with RD, and the upregulation is dependent on the form of RD. The detected proteins are present in different concentrations both in RRD and PVR. In the presence of PVR and PDR, the majority of cytokines are upregulated; thus, they may serve as biomarkers to estimate the progression or severity level of proliferation. Our study adds new biochemical information to previous studies in correlation with proliferative vitreoretinal alterations. More precise knowledge of the levels of vitreal cytokines may represent novel therapeutic targets in the management of these diseases. Future investigations should focus on identifying potential biomarkers to be able to intervene before irreversible proliferative alterations occur.

## Data Availability

The datasets generated and/or analysed during the current study are not publicly available due further analysis of data is in progress, but are available from the corresponding author on reasonable request.

## Abbreviations

CTACK: T-cell attracting chemokine
ELISA: Enzyme-linked immunosorbent assay
ERM: Epiretinal membrane
FGF: Fibroblast growth factor
G-CSF: Granulocyte colony-stimulating factor
GM-CSF: Granulocyte-macrophage colony-stimulating factor
GRO-alpha: Growth-related oncogene alpha
HGF: Hepatocyte growth factor
IFN: Interferon
IL: Interleukin
IL-1ra: Interleukin-1 receptor antagonist
IL-2Ralpha: Interleukin-2 receptor alpha
IP-10: Interferon gamma–induced protein 10
LIF: Leukaemia inhibitory factor
MCP: Monocyte chemotactic protein
M-CSF: Macrophage colony-stimulating factor
MH: Macular hole
MIF: Macrophage migration inhibitory factor
MIG: Monokine induced by interferon gamma
MIP: Macrophage inflammatory protein
beta-NGF: Beta-nerve growth factor
PDGF-BB: Platelet-derived growth factor
PDR: Proliferative diabetic retinopathy
PVR: Proliferative vitreoretinopathy
RANTES: Regulated upon activation, normal T cell expressed and secreted
RD: Retinal detachment
RRD: Rhegmatogenous retinal detachment
SCF: Stem cell factor
SDF-1alpha: Stromal cell-derived factor 1alpha
SCGF-beta: Stem cell growth factor beta
TNF: Tumour necrosis factor
TRAIL: Tumour necrosis factor-related apoptosis-inducing ligand
VEGF: Vascular endothelial growth factor
